# Freiberg’s Disease Involving First Metatarsal Bone Bilaterally in an African Male Patient: A Case Report

**DOI:** 10.7759/cureus.49093

**Published:** 2023-11-20

**Authors:** Hind El-Amin, Ahmed Mahjoub Awad Ali, Osama Khder O Elmansour, Randa Abbas, Elabbas Mohamed, Walialdeen H Biraima, Omer Kamal Ahmed, Abdallah Omer Mohamedali, Abdaljalil Arja, Ahmed O Ahmed Babikir

**Affiliations:** 1 Pain and Headache Center, Healthpoint Hospital, Abu Dhabi, ARE; 2 Surgery, Faculty of Medicine, Shendi University, Shendi, SDN; 3 Internal Medicine, Faculty of Medicine, Shendi University, Shendi, SDN; 4 General Practice, Federal Ministry of Health (Sudan), Khartoum, SDN; 5 Medicine, Faculty of Medicine, Shendi University, Shendi, SDN; 6 Internal Medicine, Federal Ministry of Health (Sudan), Shendi, SDN; 7 Pathology, Faculty of Medicine, Shendi University, Shendi, SDN

**Keywords:** atypical presentation, rare association, avascular osteonecrosis, avascular necrosis (avn), freiberg's disease

## Abstract

Freiberg's disease is a rare disorder affecting the distal metatarsal bones. With no quantitative estimate of its prevalence, the exact pathophysiology of Freiberg's disease is not clearly recognized. However, micro-trauma, repetitive injury, and vascular insufficiency have been implicated the most as predisposing factors for the condition. Freiberg’s disease typically presents in adolescent females with higher body mass index (BMI), involving the second and third metatarsal bones with an eventually destructive inflammatory process comprising swelling, hotness, tenderness, and marked restriction of movement. We report a greatly unique and highly atypical presentation of Freiberg’s disease in a middle-aged African male with bilateral and symmetrical involvement of the first metatarsal bones, a pattern of involvement that is considered highly anecdotal and atypical rarity of presentation of Freiberg's disease.

## Introduction

Freiberg's disease is a sort of avascular necrosis (AVN) affecting the distal end of metatarsal bones (the long bones of the foot). It was first described by Alfred Freiberg and colleagues in 1914, who outlined micro-traumas of the second metatarsal head as the primary etiology of the condition [1,2]. The exact pathophysiology of Freiberg's disease is not clearly recognized. The condition is considered multifactorial and is associated with the effects of multiple genes, lifestyles, and environmental factors. However, most current theories show the triggering event is predominantly traumatic (injury-related) or of a vascular insufficiency-related nature, particularly AVN [2].

Numerous cases of Freiberg's disease are asymptomatic or resolve spontaneously; thus, the prevalence of the disease is not clear. However, it is regarded as a rare disease [3,4]. The most common site of this disorder is the second metatarsal head (68%), followed by the third metatarsal (27%), and then the fourth (3%). The fifth metatarsal head is rarely involved [5]. The disease is usually unilateral and affects just a single metatarsal [4]. Bilateral involvement is rare, especially in the first metatarsal head described in the literature [6].

The diagnosis of Freiberg's disease is common during adolescence through the second decade of life [2]. The incidence is higher in females at a ratio of 3:1 [3]. Commonly, patients complain of pain and stiffness in the front of the foot, which often leads to a limp. Weight-bearing activities, including walking, generally trigger these symptoms. Signs include swelling, limited range of motion, and tenderness of the affected foot. In Freiberg's disease, early plain radiograph findings include flattening and cystic lesions of the affected metatarsal head with a widening of the metatarsophalangeal joint. In late cases, it may show osteochondral fragments, sclerosis, flattening of the bone, and increased cortical thickening. MRI typically shows flattening of the second metatarsal head, increased sclerosis, and loose body formation within the joint [7]. Histological features include a collapse of the subchondral bone, osteonecrosis, and cartilaginous fissures [3]. Most cases are self-limited. Therefore, conservative treatment, including activity modification, metatarsal pads, casting, or controlled ankle motion, and pain relief with non-steroidal anti-inflammatory medications, may be adequate, decreasing symptoms within four to six weeks. Surgery is rarely indicated [7].

## Case presentation

**Figure 1:**
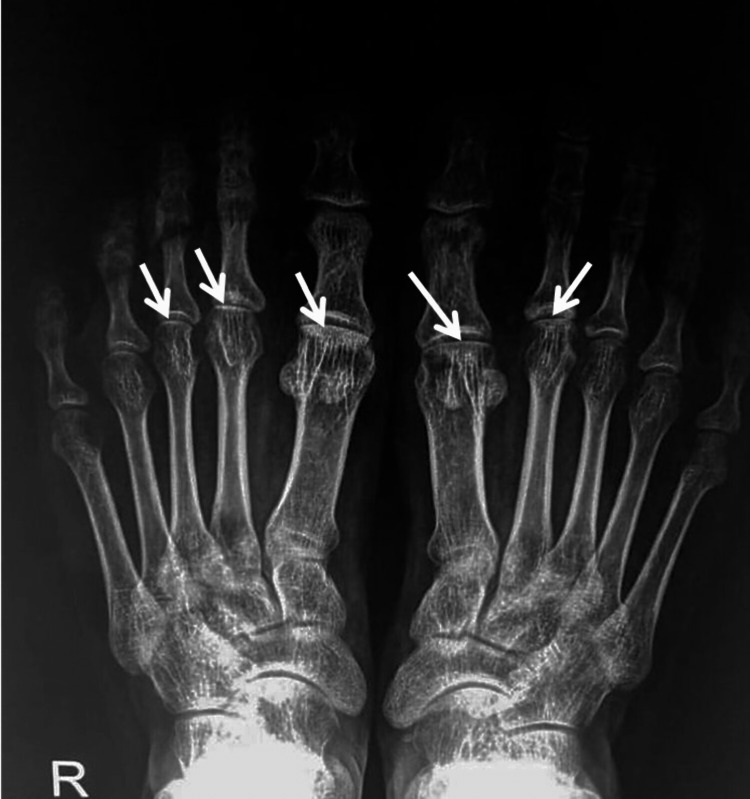
Standing X-rays of feet show flattened and sclerotic changes of the distal ends of metatarsal bones more prominently in the first metatarsal of both feet along with flattening and erosion of the articulation surfaces of the ankle joints bilaterally

A 33-year-old Sudanese male in relatively good health presented to the rheumatology clinic with a five-year history of bilateral forefoot pain and swelling, localized in the first metatarsal bone. The pain was reported as worse with standing, walking, and cold weather, and was poorly controlled with painkillers. The patient experienced a worsening in the intensity of pain two years ago with a longer duration of the attacks. This affected his quality of life, with remarkable restrictions on movement and daily activities. His condition since progressed to include his feet, ankles, and knee joints. The patient denied any history of fever or other inflammatory symptoms or signs. Physical examination revealed that he is of average build (176 cm in height, 60 kg in weight, and a BMI of 19.4) with normal vital signs. Joint examinations concluded that metatarsal joint swelling, tenderness, hotness, and restriction of movement markedly increased during dorsiflexion. X-ray radiographs taken showed flattened and sclerotic changes in the distal end of metatarsal bones, especially the first one of both feet. Also, a mild degree of flattening and erosion of the articulation surface were shown at the ankle joints (Figure 1).

**Figure 2:**
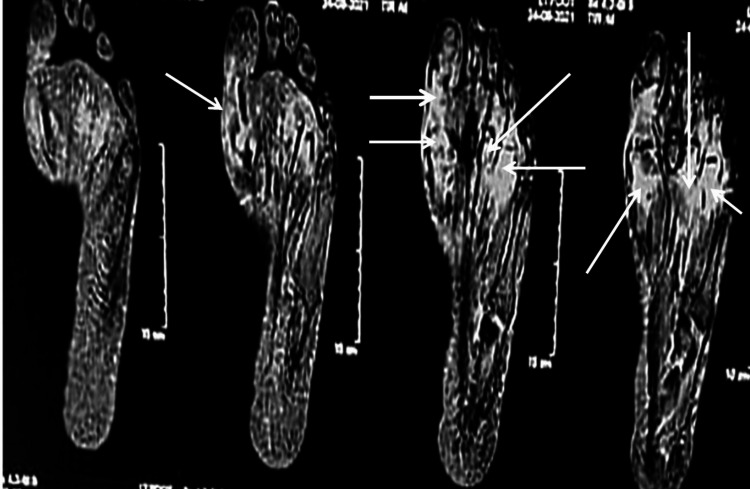
MRI shows ill-defined patchy marrow edema and associated soft-tissue edema of the head of the first, third, and fourth metatarsal bones, as well as edema of the shaft of the proximal phalanx of the fourth toe

MRI demonstrated an ill-defined patchy marrow edema signal of the head of the first, third, and fourth metatarsal bones as well as the shaft of the proximal phalanx of the fourth toe with associated soft tissue edema (Figure 2).

The diagnosis of Freiberg's disease was confirmed, and therapy with Ossofortin (Vitamin D) 20,000 IU five times per week, and pentoxifylline (a hemorheological agent) 400 mg twice daily for two months was initiated with a significant improvement in the general condition of the patient with swelling regression during the first month of treatment.

## Discussion

Freiberg's disease is most common in adolescent females; with a male:female ratio of 1:5 [1], and is associated with weight-bearing [2,6]. However, our patient was a middle-aged male with a low BMI (19.4 kg/m^2^). Clinically, most patients present with joint pain during weight-bearing, peri-articular swelling, hotness, tenderness, and restriction of movement. Being distinctive, rare, and unique, we are reporting this case to highlight the atypical presentations of the disease and to showcase its informational and educational values in adding to a better understanding of the causes of forefoot pain.

Freiberg's disease is usually unilateral. Only a few cases have been reported in which bilateral and first metatarsal bones have been affected mainly concerning female patients. As reported in the literature, flattening and sclerosis at the distal end of metatarsal bones are consistent with Freiberg's disease. This disease primarily affects the second and third metatarsal bones, representing 68% and 27% of all cases [6], respectively, but it is rare for the first metatarsal bone to be affected bilaterally, like in our case.

The clinical diagnosis of Freiberg's disease can be very challenging, considering its rarity. It can easily be misdiagnosed with other causes of metatarsalgia or connective tissue disorders. All joint disorders come with approximately the same clinical presentation (swelling, hotness, tenderness, restriction of movement) and the absence of specific signs or symptoms makes it very difficult to distinguish them. The disease comes in wide varieties from one case to another (severity, range, duration, and the number of joints involved). In this case, the atypical joint involvement and lack of a history of trauma increased the difficulty of reaching the diagnosis even more. The progression of this disease is unexpected, as it affected the ankle and knee joints too, in the current case. Based on our findings and despite trauma and impaired vascularity being considered the main causes of this disease, we would assume that autoimmunity could be connected with the pathophysiology of Freiberg's disease. It would explain the propensity for targeting the small blood vessels of the metatarsal bones or connective tissue. In addition, the increased incidence among the female population supports this hypothesis, as it's well documented that females are more prone to autoimmune disorders than males.

In the current case, the radiological findings mimicked the features of other rheumatological disorders caused by immunological reactions, e.g., rheumatoid arthritis. The previous results and the lack of a history of trauma support our argument and provoke the need to consider different clinical approaches when handling similar cases. In the future, immunological, genetic, or other factors that play a role in the progress of Freiberg's disease may be identified. Further research is needed to investigate various etiologies as well as the variable presentation of the disease.

## Conclusions

Freiberg's disease is a rare condition that primarily involves second metatarsal head ischemic necrosis. The pathology has rarely been reported to affect the other metatarsals. We report this unique case of Freiberg’s disease with a more extensive pattern and bilateral involvement in a middle-aged African male, a constellation of traits that is, to our knowledge, the first in literature. Physicians should be vigilant about such anecdotal rarities of presentation as they may pose a significant challenge to early diagnosis, management, and advancement of medical literature.
